# Regional prefrontal and hippocampal differences in gray matter volume are linked to the propensity for renewal in extinction learning

**DOI:** 10.3389/fnbeh.2025.1592929

**Published:** 2025-07-03

**Authors:** Silke Lissek, Martin Tegenthoff

**Affiliations:** Department of Neurology, BG University Hospital Bergmannsheil, Ruhr University Bochum, Bochum, Germany

**Keywords:** extinction, renewal effect, gray matter, hippocampus, anterior cingulate, inferior frontal gyrus, ventromedial prefrontal cortex

## Abstract

**Introduction:**

The renewal effect of extinction describes the reoccurrence of an extinguished response if recall is performed in a context that is not the same as the extinction context. This learning phenomenon is clinically relevant, since it potentially interferes with therapy success for anxiety disorders or phobias. The propensity to show the renewal effect appears to be a stable processing strategy in context-related extinction, associated with higher BOLD activation in hippocampus, ventromedial PFC (vmPFC) and inferior frontal gyrus (IFG) in individuals who show renewal (REN) compared to those who do not (NoREN). However, evidence on a potential relationship between structural properties such as gray matter volume (GMV) in these regions and the propensity to show renewal is lacking.

**Methods:**

In this study, we applied voxel-based morphometry (VBM) to investigate whether individuals with and without a propensity for renewal differ regarding their GMV in extinction-related brain regions, and whether such a difference is linked to the renewal level.

**Results:**

Results revealed differential GMV in REN and NoREN in adjacent subregions of IFG and vmPFC, respectively. Higher GMV in REN was located predominantly in orbital IFG and in BA10 of vmPFC. Higher GMV in NoREN was located predominantly in triangular IFG and in BA 11 of vmPFC. In bilateral anterior cingulate cortex (ACC) and anterior hippocampus, GMV was overall higher in NoREN. In the complete sample, higher GMV in IFG BA 47, vmPFC BA11, bilateral ACC and bilateral anterior hippocampus was associated with less renewal, and partially with a higher error level in extinction learning in a novel context.

**Discussion:**

The findings suggest that higher GMV in several regions active during extinction learning may support a more thorough processing of extinction trials which in turn could be conducive to an extinction recall solely based on recent extinction memory, disregarding context information. In summary, this study provides first-time evidence for a relationship of GMV in of extinction- and renewal-relevant brain regions with behavioral performance during extinction learning and the propensity to show the renewal effect.

## 1 Introduction

The renewal effect of extinction describes the reoccurrence of an extinguished response if recall is performed in a context that is not the same as the extinction context (Bouton and Bolles, 1979). In exposure therapy of anxiety disorders, fear renewal can interfere with therapy success, if successful training does not transfer from the therapeutic context to real-life situations, e.g., (Mystkowski et al., 2002). Therefore, research regarding mechanisms and neural correlates of renewal can support devising potential treatment options.

Individuals differ in their strategies of processing information during extinction learning, which reflects in their renewal rates, a behavioral measure for the propensity to show renewal. This propensity appears as an intra-individually stable, reproducible processing pattern, as found in non-fear related extinction learning (Uengoer et al., 2020). As demonstrated in fMRI studies, individuals showing renewal (REN) exhibit higher BOLD activation, indicating more intense processing, in various regions of the extinction-processing network, such as ventromedial prefrontal cortex (vmPFC), inferior frontal gyrus (IFG) and hippocampus (HC), compared to individuals who do not (NoREN) (Kalisch et al., 2006; Lissek et al., 2013; Lissek et al., 2020; Milad et al., 2007). Also anterior cingulate cortex (ACC) was found activated during non-fear related extinction learning and recall in both groups (Lissek et al., 2013). These participating regions mediate context processing (HC), context-related response inhibition and –selection (vmPFC, IFG), as well as attentional processes (ACC), respectively. The extinction networks participating in fear extinction and non-fear related extinction learning show large overlaps (Andres et al., 2024; Fullana et al., 2018; Sehlmeyer et al., 2009), e.g., vmPFC in fear extinction also processing contextual information (Gonzalez and Fanselow, 2020).

In a recent DTI study, the renewal level in REN participants was positively correlated with microstructural integrity (fractional anisotropy) in the attention-related white matter tracts of bilateral superior longitudinal fasciculus (SLF) and left cingulum (Lissek et al., 2024).

Moreover, the ability to discriminate between trials presented in a novel or identical context appears to be reduced in REN participants in bilateral IFG and posterior hippocampus, as a recent representational similarity analysis (RSA) of the representation of ABA and AAA trials in extinction-relevant brain regions showed (Lissek and Tegenthoff, 2024) - an impairment that may contribute to higher renewal.

Taken together, as yet a variety of studies suggested that processing modes associated with renewal are related to functional as well as structural properties in the brain, and have stable trait-like properties. However, it is as yet unknown whether the gray matter volumes (GMV) of extinction-relevant regions differ between individuals showing and not showing renewal.

Voxel-based morphometry (VBM) is a standardized method for voxel-wise analyses of the local gray matter in the brain (Ashburner and Friston, 2000). It is widely assumed that larger GMV is linked to superior performance, for example in executive functions (Cacciaglia et al., 2018), or in cognitive tasks (Yuan and Raz, 2014).

Research on the role of GMV in hippocampus for learning tasks yielded contradictory results, finding either no influence of GMV upon performance (Clark et al., 2020; Van Petten, 2004), or observed relationships of hippocampal GMV with the ability to rapidly learn and flexibly navigate routes (Brown et al., 2014), with the use of spatial memory strategies for a virtual maze (Bohbot et al., 2007), or with being a taxi driver (Maguire et al., 2006). It appears that GMV differences in hippocampus are predominantly related to particular spatial expertise (Woollett et al., 2008), which may also reflect context processing. Also GMV in IFG affects learning: GMV in right IFG was associated with stop signal task performance, a measure of behavioral inhibition (Wang et al., 2016). In addition, performance in a working memory task (Li et al., 2012) was associated with GMV in left IFG. GMV in anterior cingulate cortex (ACC) was increased after training of attention bias modification to threat (Carlson et al., 2022), of divergent thinking (Sun et al., 2016) and after cognitive training (Takeuchi et al., 2015). GMV in vmPFC (BA 10, BA 11) was observed to be lower in individuals who exhibited higher impulsivity (Matsuo et al., 2008). In contrast, better category shifting performance and better general executive function was found related to right vlPFC (BA 10/47) and vmPFC (BA 25/11) GMV, respectively (Smolker et al., 2015).

Only very few studies ever investigated gray matter volumes in relation to extinction learning. One study showed that the level of extinction learning (as measured by skin conductance response) was positively correlated with cortical thickness of right vmPFC (Winkelmann et al., 2016). In addition, higher cortical thickness of right vmPFC correlated with better extinction recall (Milad et al., 2005). Furthermore, vmPFC activation in extinction was positively correlated with hippocampal GMV. Besides, GMV in anterior hippocampus was larger in healthy volunteers compared to individuals with anxiety disorders (Badarnee et al., 2023). In summary, these studies support the notion that higher GMV in various brain regions strengthens the functions of these regions.

Taken together, these studies point toward a role of GMV in extinction-relevant brain regions (i.e., ACC, hippocampus, IFG and vmPFC) for processing of attention, context and response inhibition/selection, faculties that are relevant in extinction and renewal.

Since research on the relationship between GMV and the renewal effect of extinction is lacking, in this study, we investigated potential differences in GMV between individuals who show and do not show the renewal effect in a predictive learning task without a fear component. In addition, we explored the potential relationship between GMV and ABA renewal levels and extinction errors, regardless of the individual propensity for renewal. Our analysis focused on brain areas that had proved relevant for context-related extinction and renewal without a fear component in previous studies, i.e., hippocampus, IFG, vmPFC and ACC.

We assumed that REN and NoREN would differ regarding GMV in various extinction-related brain regions, and that these differences would be associated with their performance in extinction learning and recall.

## 2 Methods

### 2.1 Participants

The structural scans of 99 healthy young individuals were submitted to voxel-based morphometry analyses. These individuals had taken part in several studies using a predictive learning task to investigate context-related extinction learning and renewal without a fear component. The fMRI data and behavioral data of these studies have been previously published (Klass et al., 2017; Lissek et al., 2019; Lissek et al., 2020). Inclusion criteria for the present analysis were no treatment, or placebo treatment, in order to avoid potentially confounding effects of pharmacological treatments upon the behavioral performance, which was to be correlated to the gray matter volumes in regions of interest.

All participants were university students recruited via local advertisements at Ruhr University Bochum. Inclusion criteria for participation in the original study were: 18–40 years of age (mean age of the sample: 24.42 +/− 3.74 years), no current medical or neurological condition, no substance abuse, right-handedness, and normal or corrected-to-normal vision.

The sample analyzed here consisted of 46 men and 53 women. Based on the behavioral results from the fMRI studies, participants were assigned to two groups, according to their ABA renewal level in context-related extinction recall (see 2.6. Behavioral Data Analysis). Participants with ≤ 10% renewal were assigned to the NoREN group (total *n* = 57, men *n* = 24, women *n* = 33), participants with higher levels of renewal were assigned to the REN group (total *n* = 42, men *n* = 22, women *n* = 20).

### 2.2 Ethics statement

The studies underlying this analysis conformed to the Ethics of the Word Medical Association (Declaration of Helsinki) and were approved by the local ethics board of the Ruhr University Bochum (Reg. No. 3022-10 dated 11.02.2016). All subjects participated after giving written informed consent.

### 2.3 Image acquisition

MR images were acquired using a whole-body 3 T scanner (Philips Achieva 3.0 T X-Series, Philips, The Netherlands) with a 32-channel SENSE head coil. High-resolution structural brain images of each participant were acquired using an isotropic T1 TFE sequence (field of view 240 mm, slice thickness 1.0 mm, voxel size 1 × 1 × 1 mm) with 220 transversally oriented slices covering the whole brain. T1 images were acquired after participants performed the predictive learning task.

### 2.4 Image analysis

#### 2.4.1 VBM method

For preprocessing and statistical analysis of MR data we used the software Statistical Parametric Mapping (SPM), Version 12 (Wellcome Department of Cognitive Neurology, London, UK), implemented in Matlab R2017b (Mathworks, Natick, MA, USA). To perform voxel-based morphometry (VBM) analyses, we followed the procedure stipulated in SPM 12, which consisted of the following steps: (1) Reorienting of all datasets (orientation check of all datasets) alignment to an anterior cingulate reference. (2) Segmentation of tissues (gray matter, white matter, CSF) to generate tissue probability maps. (3) Dartel procedure to determine nonlinear transformations for warping GM and WM images for accurate intersubject alignment. (4) Normalization to standardized Montreal Neurological Institute (MNI) space using the Dartel template (voxel size 1.5 mm, FWHM 8 mm). (5) Global calculation, i.e., calculation of total intracranial volumes (TIV) to be used in the statistical analysis to prevent differences in total brain size from suggesting relevant differences in individual areas. (6) Statistics: two-sample comparisons of GM volume in REN and NoREN groups, multiple regressions of GM volume and ABA renewal level. TIV and gender were entered as covariates into the analyses.

In the statistical analyses, GMVs of extinction-relevant regions in REN and NoREN groups were compared. For these regions, anatomical ROIs were built based on the AAL atlas (anterior, posterior HC, IFG, vmPFC, anterior cingulate). The threshold for the analyses was *p* < 0.05 FWE-corrected for multiple comparisons, minimum cluster size *k* = 100 voxel, except otherwise specified.

#### 2.4.2 Regions of interest

We restricted our analyses to our *a priori* regions of interest, i.e., bilateral inferior frontal gyrus (IFG), ventromedial PFC (vmPFC) with ventrolateral PFC, hippocampus (HC) and anterior cingulate (ACC). The regions were chosen based on previous studies (Kalisch et al., 2006; Lissek et al., 2013; Lissek et al., 2018; Lissek et al., 2020; Milad et al., 2007) which showed their significant participation in extinction and renewal, by processing context features as well as response selection/inhibition and attention. For these regions we constructed bilateral anatomical ROIs based on the corresponding regions defined in the WFU pickAtlas Toolbox implemented in SPM12, using AAL atlas regions (Tzourio-Mazoyer et al., 2002).

### 2.5 Predictive learning task

The behavioral results reported below were collected during a predictive learning task performed by the participants. By means of this task, context-related extinction learning and the renewal effect of extinction without a fear component is investigated. Since the present study used the same or similar behavioral methods as a number of our prior publications among others (Lissek et al., 2013; Lissek et al., 2015; Lissek et al., 2016), we are using similar text for the task descriptions.

In the predictive learning task, originally developed by Üngör and Lachnit (2006), participants learn to associate various stimuli/cues, presented in two different contexts, with their consequences. Their responses require to state which consequence will occur to each stimulus in each trial. After each response, they receive a feedback stating the correct consequence.

The task consists of three successive phases of (a) acquisition of associations (b) extinction phase – with half of the stimuli presented in the context of acquisition (AAA condition) and the other half in a novel context (ABA condition) and (c) a test/recall phase in the context of acquisition.

**Figure 1:**
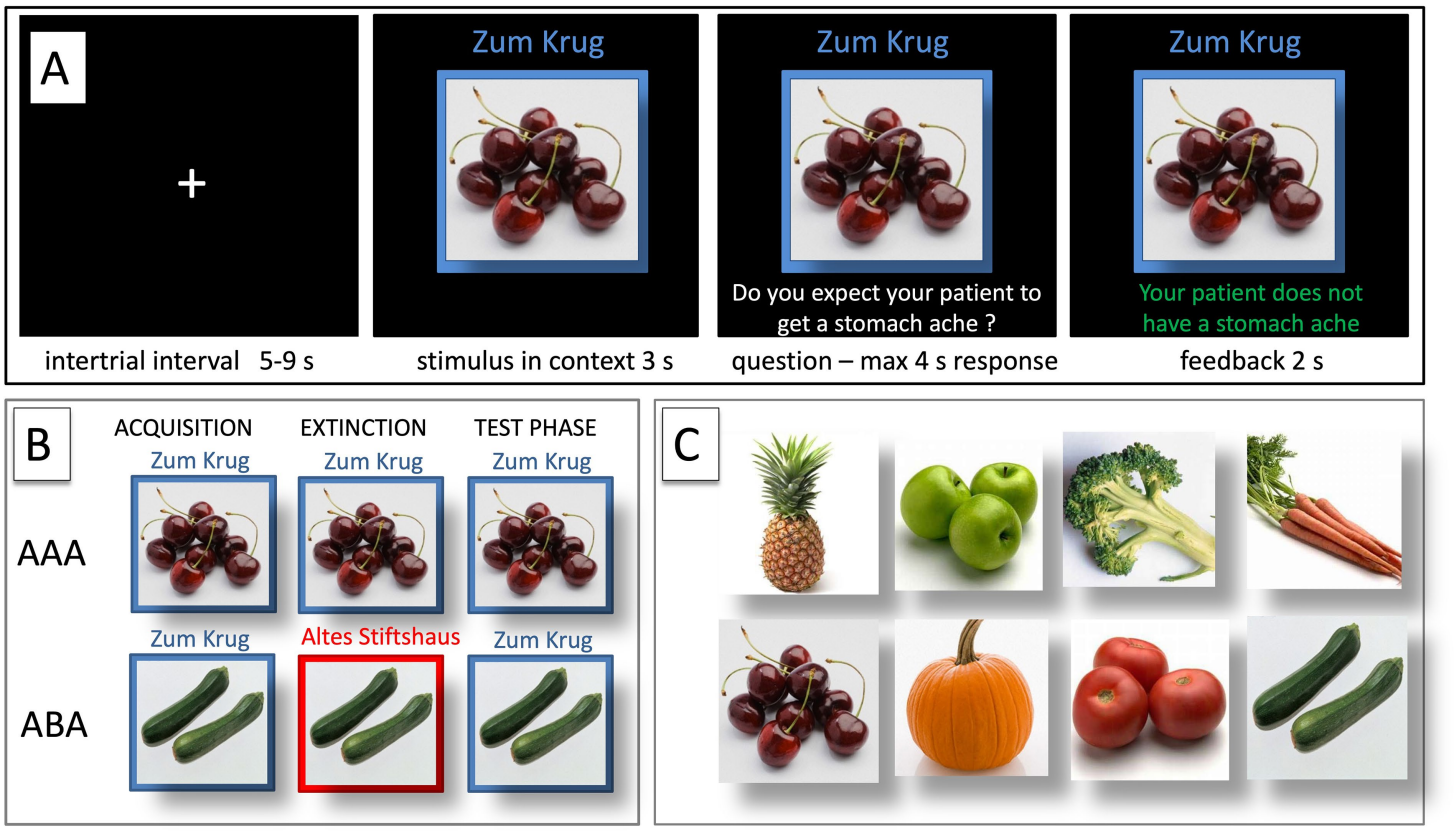
Predictive learning task. **(A)** Example of a single trial. **(B)** ABA and AAA conditions, showing the context change in ABA. **(C)** Examples of food stimuli.

During the acquisition phase, participants learn to associate a presented food item with a consequence. In each trial, a stimulus (photo of a vegetable or a fruit) is shown in one of two available contexts. The contexts consist of the restaurant names “Zum Krug” (The Mug, 1) and “Altes Stiftshaus” (The Dome, 2) and a frame in either red or blue color. (see Figure 1).

First, the stimulus in its context is presented for 3 s, then a question asking whether the patient will develop a stomach ache is added to the frame. Participants respond by pressing the respective button (“Yes” or “No”) on a keyboard within 4 s. After the response, else after expiration of the response time, feedback with the correct answer is displayed for 2 s, i.e., “The patient has a stomach ache” or “The patient does not have a stomach ache.” The actual response of the participant is not commented upon.

The food stimuli are presented in randomized order. The acquisition phase contains 16 different stimuli, eight stimuli per context. Each stimulus is presented 8 times (total 128 trials). Half of the stimuli predict stomach ache, the others no stomach ache. The consequence of stomach ache is counterbalanced to appear equally often in both contexts.

During the extinction phase, half of the stimuli from the acquisition phase (8) are presented again. Of these, one half (4) is presented in the same context as during acquisition (condition AAA—no context change) and the other half (4) in a different context (condition ABA—context change) in randomized order. These groups of stimuli are further subdivided into actual extinction stimuli (i.e., stimuli for which the consequence of stomach ache changes to no stomach ache during extinction) and retrieval stimuli (for which the consequence of stomach ache does not change), resulting in two extinction stimuli and two retrieval stimuli per context. Also, four new stimuli are introduced during the extinction phase, to balance the design so that it contains equal numbers of stimuli predicting stomach ache in both contexts. Overall, thus, the extinction phase contains a total of 12 different stimuli, six per context, i.e., two extinction stimuli, two retrieval stimuli and two new stimuli per context. Each stimulus is presented eight times (total 96 trials). In all other respects, trial design is identical to acquisition. Also, during all trial types in the extinction phase, participants receive feedback on the correctness of their response.

During the recall phase, extinction and retrieval stimuli are presented once again in the context of acquisition (five presentations per stimulus), resulting in a total of 40 trials. With the exception that during the recall phase participants receive no feedback with the correct response, trials are identical to those during acquisition.

For a detailed overview of the stimulus types, task phases, and context conditions, please refer to Table 1 and Figure 1.

**Table 1:** Task design of the predictive learning task (the classification of stimuli into extinction, retrieval, and new learning stimuli only applies from the extinction phase on).

Condition		Acquisition		Extinction		Test	
		Context 1	Context 2	Context 1	Context 2	Context 1	Context 2
AAA	Extinction	A+	B+	A-	B-	A?	B?
	Retrieval	C+	D-	C+	D-	C?	D?
	New learning	I- Q-	J- R+	K+	L+		
ABA	Extinction	E+	F+	F-	E-	E?	F?
	Retrieval	G+	H-	H-	G+	G?	H?
	New learning	M- S-	N- T+	P+	O+		

Extinction stimuli and Retrieval stimuli appeared in all learning phases. New learning stimuli appeared only in the respective learning phase to balance the design. Overall, + and – signs after the stimuli letters indicate whether the stimulus signaled stomach ache (= CS+) or no stomach ache (= CS-).

### 2.6 Behavioral data analysis

We calculated the percentage of extinction errors in the ABA and AAA conditions in the extinction learning phase, as well as the level of ABA renewal during the recall phase.

Errors during the extinction phase are subdivided into ABA extinction errors and AAA extinction errors for trials in the respective conditions.

The level of ABA renewal is defined by the percentage of responses in ABA trials of the recall phase, in which the response is given that was correct during acquisition.

The data were derived from log files written for all three learning phases, from which we calculated error rates during extinction learning. For calculation of the renewal level, during the recall phase only responses to stimuli with consequence change (extinction stimuli) were analyzed. The behavioral renewal effect in the predictive learning task is supposed to occur only in the condition ABA, due to the context change introduced during extinction learning. In case of renewal, associations learned during acquisition in context A will reflect in responses during the test phase, which is again performed in context A, while extinction was performed in context B. In contrast, the AAA condition constitutes a control condition for extinction learning, since here all learning phases are performed in an identical context. If extinction learning is successful, responses during the test phase will reflect the associations learned during the extinction phase. However, if extinction learning is impaired, responses in the AAA test phase may reflect associations learned during acquisition.

Errors in acquisition and extinction learning were defined as responses stating the incorrect association between the context-cue-compound and the consequence.

During the recall phase, a response that referred to the association which was correct during acquisition constituted an error in the AAA condition, and a renewal response in the ABA condition. The ABA renewal level was calculated as the percentage of renewal responses in all ABA trials during the recall phase.

Statistical analyses were performed using the software package IBM SPSS Statistics for Windows, version 27.0 (IBM Corp, Armonk, NY, United States).

## 3 Results

### 3.1 Behavioral performance data

By definition, REN and NoREN groups differed with regard to their respective ABA renewal levels (*F*(1) = 258.538 *p* < 0.001). With regard to extinction learning performance, there were no significant differences between the groups in ABA extinction errors (*F*(1) = 0.072 *p* = 0.789). Also in AAA extinction trials, the groups did not differ significantly, even though the REN group made more errors than the NoREN group (*F*(1) = 2.961 *p* = 0.088) (Table 2).

**Table 2:** Behavioral performance of the REN and NoREN groups in the predictive learning task, renewal and error rates in percent (+/− standard error of means).

Subgroup	ABA renewal in percent +/− s.e.m.	ABA extinction errors in percent +/− s.e.m.	AAA extinction errors in percent +/− s.e.m.
REN	61.55 +/− 4.45	16.25 +/− 1.74	20.40 +/− 1.90
NoREN	0.18 +/− 0.18	16.94 +/− 1.79	16.32 +/− 1.48
	*F*(1) = 258.538 *p* < 0.001	*F*(1) = 0.072 *p* = 0.789	*F*(1) = 2.961 *p* = 0.088

A multivariate ANOVA revealed no significant gender differences in ABA extinction learning (*F*(1) = 0.235 *p* = 0.629; men: 17.31% +/−1.82 sem; women: 16.07% +/− 1.77 sem), or AAA extinction learning (*F*(1) = 0.956 *p* = 0.331; men: 19.29% +/− 2.00 sem; women: 16.97% +/− 1.37 sem), nor in ABA renewal (*F*(1) = 0.116 *p* = 0.734; men: 24.89% +/− 4.77 sem; women: 27.36% +/− 5.31 sem).

### 3.2 GMV comparisons of REN and NoREN participants

**Figure 2:**
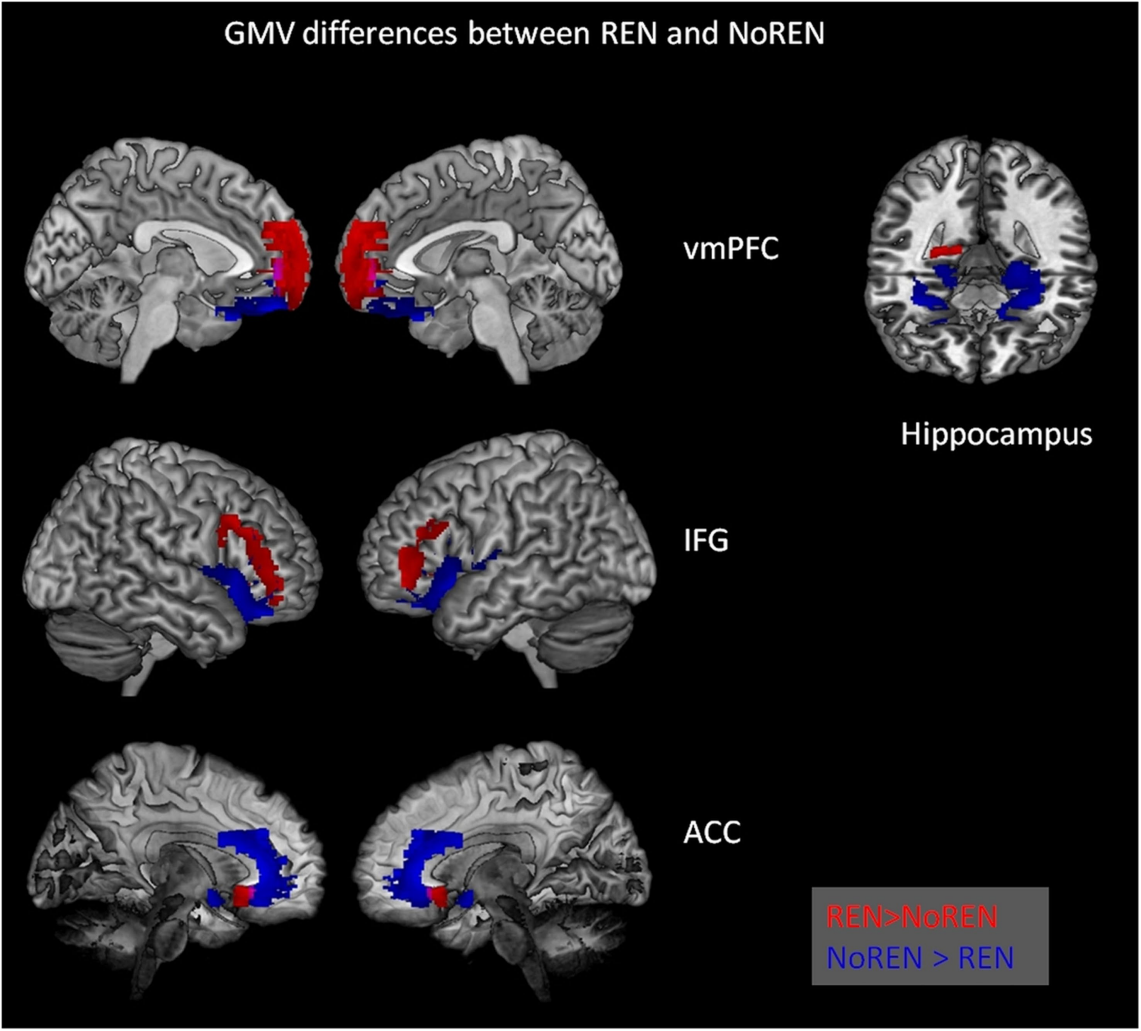
Significant differences in gray matter volume between REN and NoREN individuals. Red indicates areas where REN has higher GMV than NoREN, Blue indicates regions where NoREN has higher GMV than REN. Two-sample *t*-tests, threshold FWE *p* < 0.05 *k* = 100 voxel, corrected for TIV, covariate gender.

In SPM, we calculated contrasts to compare REN and NoREN participants with regard to their GMV in a number of extinction-relevant brain regions (anterior and posterior hippocampus, vmPFC, IFG, and ACC) selected as *a priori* ROIs. The reported results are corrected for total intracranial volume (TIV) and contain the covariate gender (see Table 3 and Figure 2).

**Table 3:** (a) Regions with higher GMV in REN than in NoREN. (b) Regions with higher GMV in NoREN than in REN, threshold FWE *p* < 0.05 *k* = 100 voxel, corrected for TIV, covariate gender.

MNI peak coordinate	Region	*t*-value	*p*-value	Voxel	Effect size (Cohen’s d)
(a) REN > NoREN					
24 36 −8	Right orbital/triangular IFG BA 47	9.12	0.000	353	1.85
−51 47 −9	Left triangular/orbital IFG BA 46/47/45	8.41	0.000	689	1.65
−29 35 −5	Left orbital IFG BA 47	7.62	0.000	338	1.55
59 23 35	Right triangular/orbital IFG BA 46/9/47/45	8.11	0.000	1,429	1.65
36 54 29	Right middle frontal gyrus, medial/lateral BA 10	10.66	0.000	3,075	2.17
−26 62 24	Left middle frontal gyrus, medial/lateral BA 10	9.96	0.000	2,961	2.02
12 26 −2	Right anterior cingulate BA 25	7.38	0.000	147	1.50
−11 24 −2	Left anterior cingulate BA 25	6.80	0.000	108	1.38
(b) NoREN > REN					
33 44 −21	Right triangular/orbital IFG BA 47/45	11.43	0.000	3,508	2.32
−18 23 −27	Left triangular/orbital/opercular IFG BA 47	9.95	0.000	3,693	2.02
−36 26 27	Left triangular IFG	9.91	0.000	198	2.01
41 30 26	Right triangular IFG. BA 46	9.45	0.000	263	1.92
−12 36 −35	Left superior frontal gyrus orbital part BA 11	11.93	0.000	964	2.43
24 45 −24	Right middle frontal gyrus orbital part BA 11	13.56	0.000	1,147	2.76
29 49 −2	Right superior frontal gyrus orbital part BA 10	10.78	0.000	129	2.19
−24 48–6	Left superior frontal gyrus orbital part BA 10/11	8.64	0.000	116	1.76
38 −8 −21	Right anterior hippocampus	7.42	0.000	730	1.51
−14 −2 −14	Left anterior hippocampus	6.42	0.000	1,371	1.31
24 −32 −2	Right posterior hippocampus	4.99	0.000	107	1.01
12 36 8	Bilateral anterior cingulate BA 24/32/33	9.15	0.000	3,762	1.86
−5 2 −5	Left anterior cingulate BA 25	7.34	0.000	178	1.49

In inferior frontal gyrus, adjacent areas showed either higher GMV in REN or in NoREN. Also in the vmPFC area, regions predominantly in BA 10 showed higher GMV in REN, while other regions in BA 11 showed higher GMV in NoREN. In contrast, in both anterior and posterior hippocampus, there were only clusters with a higher GMV in NoREN than in REN, but not vice versa. We observed several large clusters in bilateral anterior hippocampus and a small one in right posterior hippocampus. In anterior cingulate, NoREN showed large clusters of higher GMV, compared to only small clusters of higher GMV in REN.

### 3.3 Multiple regression: relationship of GMV and ABA renewal level

**Figure 3:**
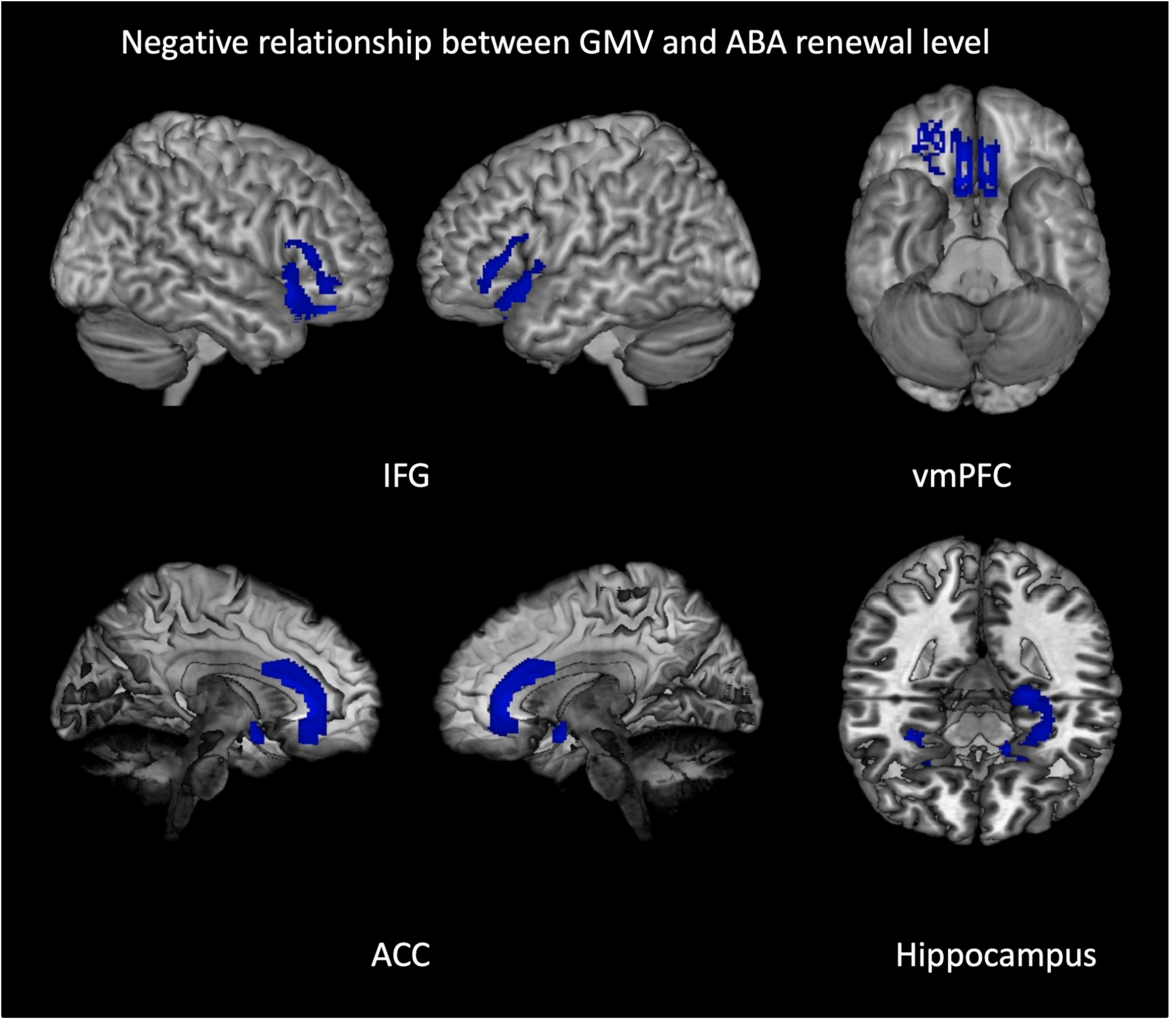
Negative relationship between gray matter volume and ABA renewal in the complete group (REN and NoREN participants). Multiple regression analysis, covariates gender and TIV, threshold FWE *p* < 0.05 *k* = 100. The figure shows clusters with a negative relationship between gray matter volume and ABA renewal in the complete group (REN and NoREN participants), indicating that higher GMV in these regions was associated with a lower level of ABA renewal. Multiple regression analysis containing gender and TIV as covariates, threshold FWE *p* < 0.05 *k* = 100.

**Figure 4:**
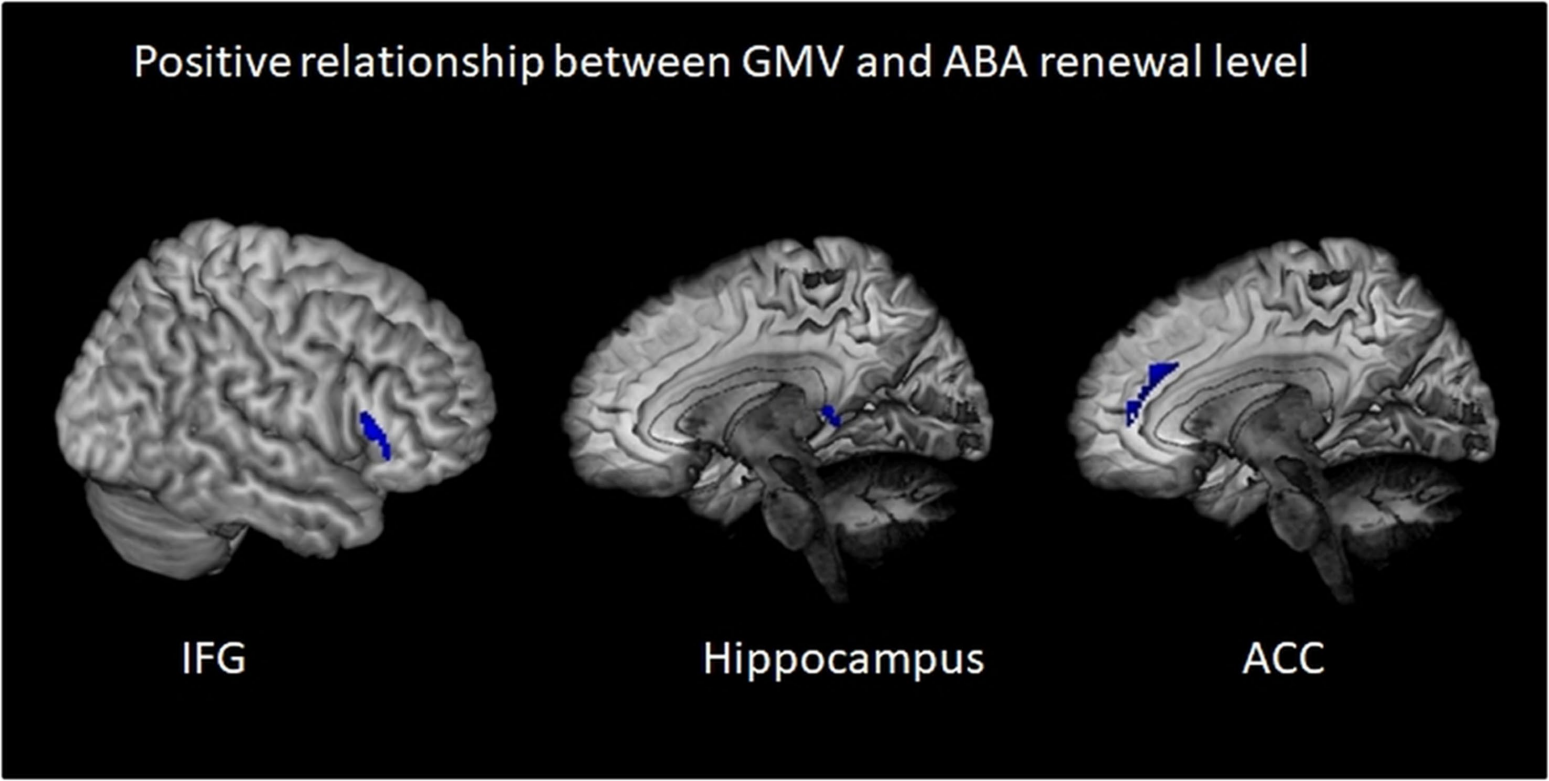
Positive relationship between gray matter volume and ABA renewal in the complete group (REN and NoREN participants). Multiple regression analysis, covariates gender and TIV, threshold for IFG result: FWE *p* < 0.05 *k* = 100, for HC and ACC result: *p* < 0.05 uncorr., *k* = 100. The figure shows clusters with a negative relationship between gray matter volume and ABA renewal in the complete group (REN and NoREN participants), indicating that higher GMV in these regions was associated with a higher level of ABA renewal.

We calculated multiple regressions to evaluate the relationship of the ABA renewal level with GMV in a number of extinction-relevant brain regions (hippocampus, ventromedial PFC, inferior frontal gyrus, and anterior cingulate) for the complete sample. The reported results are corrected for total intracranial volume (TIV) and contain the covariate gender. The relationship between GMV and renewal is predominantly negative, particularly in bilateral anterior hippocampus, but also in bilateral IFG and ACC and in vmPFC (see Table 4 and Figure 3). In contrast, in some substantially smaller clusters, also a positive relationship was observed: in right triangular/orbital IFG and right posterior hippocampus, as well as in right anterior cingulate. For the latter two, the relationship was significant only when using an uncorrected threshold (see Table 5 and Figure 4).

**Table 4:** A negative relation between GMV and renewal level in the complete group (containing all REN and NoREN participants) was observed in the following regions (threshold FWE *p* < 0.05 *k* = 100 voxel, corrected for TIV, covariate gender).

MNI peak coordinate	Region	*t*-value	*p*-value	Voxel	Effect size (Cohen’s d)
33 44 −20	Right orbital IFG BA 47	7.32	0.000	871	0.74
−18 21 −27	Left orbital/opercular IFG BA 47	6.90	0.000	247	0.69
47 32 9	Right triangular/opercular IFG BA 47	5.87	0.000	317	0.59
−47 20 −12	Left orbital IFG BA 47	6.05	0.000	319	0.61
−44 35 0	Left triangular IFG BA 47	5.77	0.000	366	0.58
8 20 −29	Right orbital frontal lobe gyrus rectus. BA 11	7.93	0.000	308	0.80
−5 21 −29	Left orbital frontal lobe gyrus rectus BA 11	7.11	0.000	269	0.71
24 45 −21	Right superior frontal gyrus BA 11	6.93	0.000	179	0.70
−14 −2 −14	Left anterior hippocampus	5.57	0.000	720	0.56
35 −8 −20	Right anterior hippocampus	5.17	0.000	198	0.52
11 36 6	Bilateral Anterior cingulate BA 24/33/32	5.96	0.000	1,518	0.60

**Table 5:** A positive relation between GMV and renewal level in the complete group (containing all REN and NoREN participants) was observed in the following regions (threshold FWE *p* < 0.05 *k* = 100 voxel unless otherwise specified (*), corrected for TIV, covariate gender).

MNI peak coordinate	Region	*t*-value	*p*-value	Voxel	Effect size (Cohen’s d)
45 24 6	Right triangular/orbital IFG BA 13/47	4.94	0.002	104	0.50
23 −38 6	Right posterior hippocampus*	4.36	0.000	118	0.44
12 36 27	Right anterior cingulate *	5.35	0.000	124	0.54

*p < 0.05 uncorrected, k = 100.

The negative relationship indicates that higher GMV in these regions is associated with less renewal. Thus it can be expected that regions showing up in this analysis correspond largely to the regions showing up in the above two-sample comparison of NoREN > REN. This is actually the case for: bilateral IFG BA 47, right BA 11, bilateral anterior hippocampus, and bilateral anterior cingulate.

### 3.4 Multiple regression: relationship of GMV and ABA renewal level and ABA extinction errors

In this multiple regression analysis, we introduced an additional covariate of interest, namely ABA extinction errors, in addition to ABA renewal level. This analysis was performed to identify the regions in which both these parameters were simultaneously related to GMV. Results here are reported using an uncorrected threshold of *p* < 0.01.

**Figure 5:**
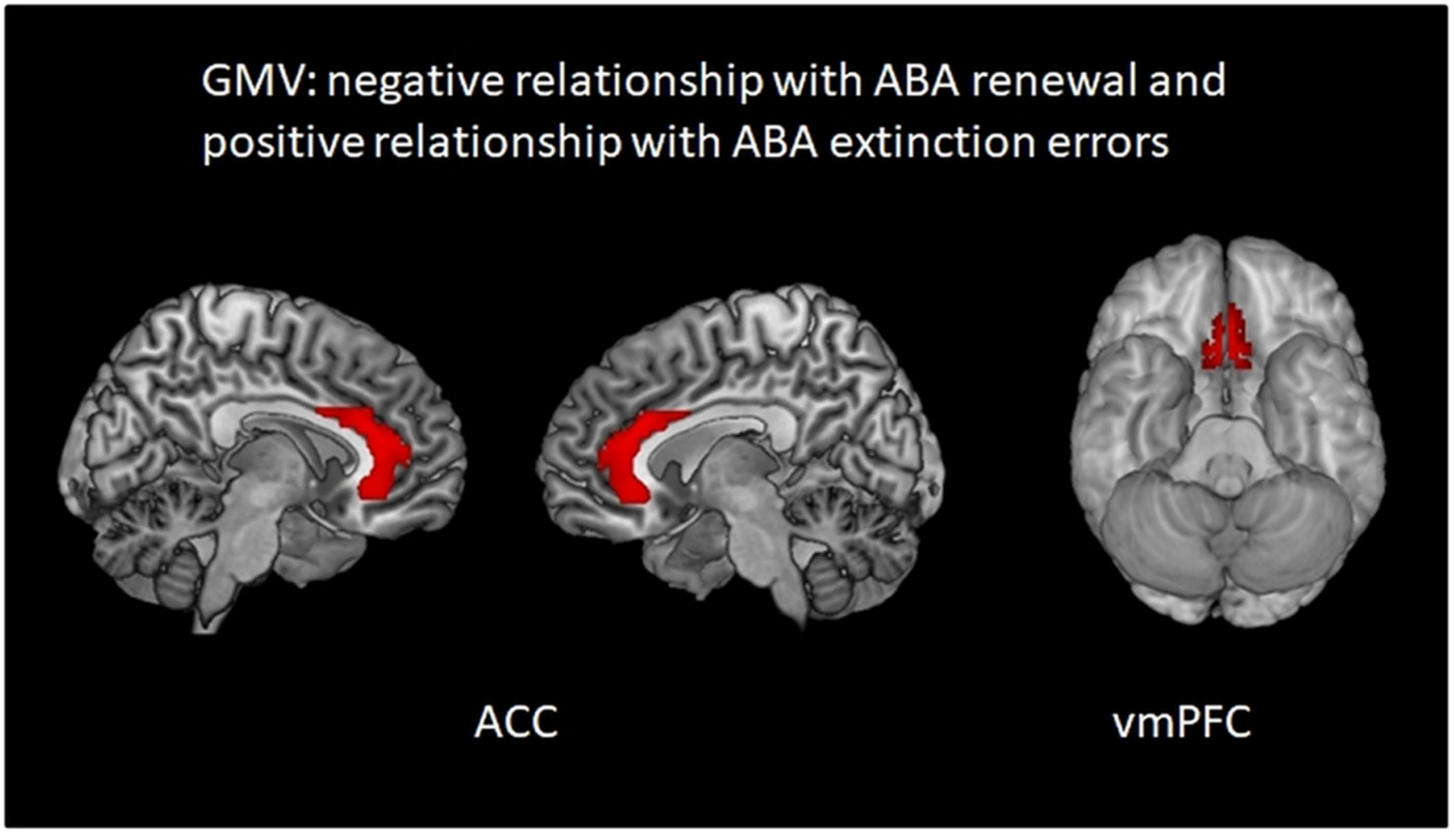
Regions in which higher GMV is associated simultaneously with less ABA renewal and more ABA extinction errors: areas in bilateral ACC and vmPFC. Multiple regression analysis, covariates gender and TIV, threshold *p* < 0.01 uncorrected *k* = 100.

A negative relationship of GMV with ABA renewal level together with a positive relationship with ABA extinction errors was found in bilateral vmPFC BA 11 and bilateral ACC. The results indicate that in these regions high GMV was linked to slower ABA extinction and subsequently less ABA renewal. Vice versa, lower GMV here was linked to faster ABA extinction and subsequently to more ABA renewal (see Table 6 and Figure 5).

**Table 6:** GMV relationship with ABA renewal level (negative) and ABA extinction errors (positive) in the complete group (containing REN and NoREN participants) threshold *p* < 0.01 uncorrected *k* = 100 voxel, corrected for TIV, covariate gender.

MNI peak coordinate	Region	*t*-value	*p*-value peak	Voxel	Effect size (Cohen’s d)
−2 33 −29	Left BA 11	4.19	0.000	354	0.42
3 21 −27	Right BA 11	4.03	0.000	251	0.41
12 19 23	Bilateral anterior cingulate	3.39	0.001	3,059	0.34

## 4 Discussion

In this study, we compared GMV between REN and NoREN individuals in extinction-relevant brain regions. We found significant GMV differences in several regions, which were partially associated with ABA renewal rates and extinction errors.

- In adjacent subregions of IFG and vmPFC, GMV differed between REN and NoREN.
- In IFG, higher GMV in REN was located predominantly in orbital IFG, in NoREN in triangular IFG.
- In vmPFC, higher GMV in REN was observed predominantly in BA 10, in NoREN predominantly in BA 11.
- In anterior hippocampus as well as in bilateral ACC, GMV was overall higher in NoREN.
- In the complete sample, higher GMV in IFG BA 47, vmPFC BA11, bilateral ACC and bilateral anterior hippocampus was associated with less ABA renewal (and partially with a higher error level in ABA extinction).

### 4.1 Double dissociation of higher GMV in vmPFC in REN and in NoREN

Higher GMV in NoREN compared to REN was found in BA 11 of vmPFC, and accordingly, higher GMV in this region was associated with less ABA renewal.

In contrast, in BA 10 of vmPFC, GMV was predominantly higher in REN participants. This finding of differential GMV extends previous findings of REN showing more pronounced BOLD activation in BA 10 during ABA trials in the recall phase, in which renewal occurs (Lissek et al., 2013). Also in studies that investigated effects of various neurotransmitters upon renewal, vmPFC BA 10 was found more active in REN participants (Klass et al., 2021).

Thus, the double dissociation of higher GMV in vmPFC regions of BA 10 and BA 11 in REN and NoREN individuals may point toward differential processing proclivities in these two groups which could have an impact upon the tendency to show renewal.

A dissociation of responding in BA 10 and BA 11 of vmPFC was also observed in a study that investigated reversal learning in humans (Zhang et al., 2015) and found differences in the focus of processing: activation in bilateral medial BA 11 was associated predominantly with response inhibition of a previously learned, and presently obsolete, response; however, activation in left medial BA 10 was positively correlated with reward magnitude, i.e., focussing on the outcome.

If, in our task, higher GMV in BA 11 – as observed in NoREN - supports better inhibition of the response learned during acquisition, regardless of context, such processing may result in less ABA renewal. In contrast, higher GMV in BA 10 – as observed in REN -, which according to the above study might support focussing on an outcome, may - combined with a contextual association of the outcome - rather favor ABA renewal.

However, these findings refer predominantly to medial BA 10, while the higher GMV in REN was found in medial and lateral BA 10. In a recent paper describing a model of four streams in the prefrontal cortex, the authors (Ben Shalom and Skandalakis, 2024) deliver an account of studies that show which areas of BA 10 and BA 11 (among others) are involved in certain processing tasks. In their account, medial BA 10 activation may reflect integration of prior knowledge and likelihood (Ting et al., 2015), while lateral BA 10 higher activation is apparently associated with higher demands on perceptually detailed information (Ranganath et al., 2000) as well as switching between present and previously encoded memory episodes (Koechlin and Summerfield, 2007). In contrast, medial BA 11 is assumed to be related to combining prior and current sensory information (Chaumon et al., 2014), and lateral BA 11 in updating sensory information and selecting rewarded stimuli.

Projecting these findings onto the results of our study, REN with their higher GMV in lateral BA 10 may have a processing focus upon perceptual details, such as the different contexts. Thus, REN may be able to easier switch between the responses learned during acquisition and extinction, making the response that was correct in acquisition more accessible, which would favor ABA renewal.

### 4.2 Regions in IFG show higher GMV in REN and NoREN, respectively

In the subregions of IFG (orbital, triangular, opercular), several adjacent areas displayed higher GMV either in NoREN or in REN. Overall, though, areas in which NoREN showed higher GMV were considerably more extended. Of note, higher GMV in BA 47 was associated with less ABA renewal, suggesting that this finding is based largely on the NoREN data.

Complementing previous results, the rather dorsally located IFG regions of higher GMV in REN that we found in the present analysis correspond to peak voxel findings of studies reporting IFG BOLD activation in REN individuals relative to NoREN. For example, in a study comparing activation to extinction trials with more or less salient contexts (Lissek et al., 2020), areas of higher activation in IFG were located rather dorsally in REN, and rather ventrally in NoREN – suggesting that differences in GMV may have had a role in that pattern of activation in the subgroups.

The higher GMV of NoREN in BA 47 of IFG may also support their performance, since this region is involved in response inhibition (Hampshire et al., 2010; Swick et al., 2008), and in response selection between competing response options (Budhani et al., 2007; Mitchell et al., 2009). Moreover, context-related discrimination of stimuli in recall (e.g., discrimination between ABA and AAA stimuli) was recently found linked to ABA extinction errors in BA 47 in both REN and NoREN, suggesting that this type of discrimination supports response selection in the recall phase (Lissek and Tegenthoff, 2024). Thus, higher GMV in IFG BA 47 may support better response inhibition for extinction stimuli, which in turn may support a processing mode that does not result in renewal.

In addition, in the present study, GMV in triangular IFG / BA 45 was higher in NoREN. In the same region, NoREN individuals of the above-mentioned study (Lissek and Tegenthoff, 2024), showed superior discrimination performance for extinction and retrieval trials (during recall). Further studies found BA 45 involved in response inhibition too (Adelhöfer and Beste, 2020; Boecker et al., 2011; Collette et al., 2001; Rubia et al., 2003), which suggests that higher GMV in triangular IFG may also boost response inhibition performance.

In summary, the higher GMV of NoREN in IFG regions involved in response inhibition suggests superior processing capabilities, which may contribute to the absence of renewal in this group.

### 4.3 Higher GMV in anterior HC in NoREN

In bilateral anterior HC, GMV was higher in NoREN individuals compared to REN. According to the literature [e.g., (Brown et al., 2014; Koch et al., 2016)], it can be assumed that the higher GMV enhanced anterior HC-based processing.

There is evidence that anterior hippocampus is involved in processing, for example, novel compared to familiar scenes (Poppenk et al., 2010), alterations of the spatial configuration of a scene (Howard et al., 2011), or recall of past experiences, e.g., (Hassabis et al., 2007). Taken together, it has been argued that anterior HC may be recruited when constructing a scene from representations that are distributed across the cortex (novel or consolidated memories) (Zeidman et al., 2015). In the case of the predictive learning task, therefore, anterior HC may combine stimulus, context(s) in which the stimulus appeared, and the respective outcomes.

A higher GMV in anterior HC, as found in this study for NoREN participants, may thus also be a cause of differences in stimulus discrimination ability that we observed previously: in NoREN, but not in REN individuals, context- and outcome-related discrimination ability in anterior hippocampus during the recall phase was linked to the number of AAA extinction errors (Lissek and Tegenthoff, 2024) in the preceding extinction learning phase. Thus, in NoREN, anterior hippocampus may play a role in using prediction error information during recall, with this better processing ability potentially linked to their higher GMV.

Interestingly, healthy controls differ from anxiety patients with regard to GMV of anterior HC (Badarnee et al., 2023), with anxiety patients exhibiting lower GMV in anterior HC than controls. This is particularly interesting in view of the finding that persons with anxiety disorders also tend to show impaired (fear) extinction learning (Duits et al., 2015; Duits et al., 2016). From these findings, showing renewal could be associated with an impairment in extinction learning due to suboptimal stimulus discrimination ability, in which a lack of processing in anterior hippocampus may be involved.

Moreover, higher anterior HC activation was previously found in NoREN compared to REN during the initial presentation of a task context (Lissek et al., 2016), presumably related to novelty processing, another function of this region (Poppenk et al., 2010). In contrast, REN preferentially activated posterior HC during this phase, suggesting differential hippocampal processing of novel information in REN and NoREN already during initial learning, which may influence later processing. The present results expand these previous findings, suggesting that the activation and processing differences observed between NoREN and REN in anterior HC may be linked to the higher GMV volume in NoREN in this region.

In summary, the present results of higher GMV in anterior HC of NoREN individuals indicate that they may have more potential to process novel (context) information, possess better context-related stimulus discrimination ability, and make better use of prediction error information in extinction learning.

### 4.4 Higher GMV in bilateral ACC in NoREN

Also, GMV in a large region of bilateral anterior cingulate (BA 24, 32, 33, 25) was higher in NoREN. In contrast, only two small clusters in bilateral ACC in BA 25 showed higher GMV in REN.

A higher GMV in ACC implicates better attentional processing (Carlson et al., 2022), which in synergy with ongoing processing in other brain regions may support recall of extinction memory regardless of context. In line with this, ACC appears to be involved in computation of prediction errors (Vassena et al., 2017), and thus may support processing of extinction errors. In our study, a higher rate of ABA extinction errors may provide more “learning material” to a proficient ACC, and may thus contribute to better extinction recall, i.e., lower ABA renewal. Accordingly, higher GMV in the NoREN cluster was found associated with less ABA renewal and more ABA extinction errors.

In a previous study we also observed higher BOLD activation in ACC to ABA stimuli than to AAA stimuli during extinction recall in both groups (Lissek et al., 2013), corresponding to the observations presented above. Also, this activation was higher in NoREN subjects than in REN subjects, not only during the recall phase, but also during extinction learning proper. This higher activation could potentially be related to the higher GMV in NoREN, considering evidence that in a cognitive task, gray matter volume and BOLD activation can be positively correlated (Kannurpatti et al., 2010).

In summary, the present results suggest that higher GMV in ACC may strengthen attentional processing of prediction errors and thus promote better extinction recall in the NoREN group.

### 4.5 Higher GMV in several regions correlated with more ABA extinction errors together with fewer ABA renewal responses

Interestingly, in the complete group of all REN and NoREN individuals, higher GMV in specific clusters in IFG BA 47, vmPFC BA11, bilateral ACC and bilateral anterior hippocampus was associated with fewer ABA renewal responses. As can be expected, these regions widely overlapped with those identified in the two-sample comparison NoREN versus REN regarding GMV, i.e., with clusters showing higher GMV in NoREN, and thus support the notion of a relationship between more gray matter in several extinction-relevant brain regions and less renewal.

As mentioned above in discussing higher GMV in the individual brain regions, the diverse functions of vmPFC BA 11, IFG BA 47, as well as bilateral ACC and anterior hippocampus, which all contribute to successful extinction learning, in the NoREN group may have been supported by their higher GMV, enabling the observed lack of ABA renewal.

Using a less conservative threshold of significance, we also observed a link between higher GMV in both vmPFC BA 11 and bilateral ACC with fewer ABA renewal responses and, simultaneously, more errors in the preceding ABA extinction phase. These findings suggest that the amount of errors in ABA extinction may have the potential to influence the level of ABA renewal, and that processing of both is probably related to higher GMV in these regions. A higher error level in ABA extinction trials may compel more attention to the stimuli in question, and therefore may have been conducive to more elaborate processing and subsequent better encoding of correct responses to these stimuli. Such a better encoding may consequently have led to reduced or non-existent ABA renewal.

Vice versa, lower GMV in these regions was linked to faster ABA extinction and subsequently to more ABA renewal – suggesting that individuals with lower GMV altered their response more quickly and, therefore, did not experience as many episodes of a wrong response to process and encode, which may have led to the perpetuation of the initially correct but subsequently obsolete response into the recall phase.

Following this line of argumentation, it can be speculated that more efficient processing - mediated by high GMV in these regions – consists of (a) stronger resistance against extinction when it occurs in a novel context, combined with (b) disregarding the context during the recall phase, focussing instead only on the altered contingency between stimulus and response. The performance pattern suggests that the novel context first causes some confusion and is later discarded as irrelevant.

Vice versa, less efficient processing – mediated by lower GMV in these regions – consists of (a) easier, quicker extinction that presumably uses context as an important cue, so that (b) the context is integrated in the stimulus–response association which thus leads to an ABA renewal response in the recall phase.

### 4.6 Limitations of the study

The present findings on the relationship of GMV in extinction-related brain regions and renewal are based on a non-fear-related learning task, and therefore may not readily translate to fear renewal, which bears clinical relevance by potentially impeding the therapy success of exposure therapies that are based on fear extinction learning. Even though extinction procedures with and without a fear component largely recruit a similar network of extinction-relevant regions, the differences between the procedures of fear extinction and our predictive learning task preclude speculations about the meaning of our present findings for clinical treatment.

Future research should therefore investigate GMV in the extinction network in relation to results of fear renewal tasks. Another option would be to compare men and women with regard to GMV and renewal, to determine whether gender differences exist in the participating processing regions.

## 5 Conclusion

To the best of our knowledge, this study is the first to systematically investigate the relationship of GMV in extinction-relevant brain regions with behavioral extinction criteria, i.e., extinction error rates and renewal rates. The results demonstrate significant GMV differences between individuals with and without a propensity for renewal. A double dissociation with higher GMV in BA 11 in NoREN and higher GMV in BA 10 in REN characterizes vmPFC findings, while in adjacent regions of IFG, GMV is higher in either REN or NoREN. In general, higher GMV was found associated with a lower renewal rate, and in several regions also with a higher extinction learning error rate for trials presented in a novel context. Thus, the findings suggest that higher GMV may support a more thorough processing of extinction trials which in turn could be conducive to an extinction recall solely based on recent extinction memory, disregarding context information. These findings shed additional light upon the relationship of brain structural properties with processing of extinction learning.

## Data Availability

The raw data supporting the conclusions of this article will be made available by the authors, without undue reservation.

## References

[ref1] Adelhöfer N. Beste C. (2020). Pre-trial theta band activity in the ventromedial prefrontal cortex correlates with inhibition-related theta band activity in the right inferior frontal cortex. Neuroimage 219:117052. doi: 10.1016/j.neuroimage.2020.117052, PMID: 32540357

[ref2] Andres E. Meyer B. Yuen K. S. L. Kalisch R. (2024). Current state of the neuroscience of fear extinction and its relevance to anxiety disorders. Curr. Top Behav. Neurosci. 3, 1–20. doi: 10.1007/7854_2024_555 39747796

[ref3] Ashburner J. Friston K. J. (2000). Voxel-based morphometry - the methods. Neuroimage 11, 805–821. doi: 10.1006/nimg.2000.0582, PMID: 10860804

[ref4] Badarnee M. Wen Z. Nassar N. Milad M. R. (2023). Gray matter associations with extinction-induced neural activation in patients with anxiety disorders. J. Psychiatr. Res. 162, 180–186. doi: 10.1016/j.jpsychires.2023.05.015, PMID: 37167838

[ref5] Ben Shalom D. Skandalakis G. P. (2024). Four streams within the prefrontal cortex: integrating structural and functional connectivity. Neuroscientist 31, 8–13. doi: 10.1177/10738584241245304, PMID: 38577969 PMC11809115

[ref6] Boecker M. Drueke B. Vorhold V. Knops A. Philippen B. Gauggel S. (2011). When response inhibition is followed by response reengagement: an event-related fMRI study. Hum. Brain Mapp. 32, 94–106. doi: 10.1002/hbm.21001, PMID: 20336654 PMC6870390

[ref7] Bohbot V. D. Lerch J. Thorndycraft B. Iaria G. Zijdenbos A. P. (2007). Gray matter differences correlate with spontaneous strategies in a human virtual navigation task. J. Neurosci. 27, 10078–10083. doi: 10.1523/JNEUROSCI.1763-07.2007, PMID: 17881514 PMC6672675

[ref8] Bouton M. E. Bolles R. C. (1979). Role of conditioned contextual stimuli in reinstatement of extinguished fear. J. Exp. Psychol. Anim. Behav. Process. 5, 368–378. doi: 10.1037/0097-7403.5.4.368, PMID: 528893

[ref9] Brown T. I. Whiteman A. S. Aselcioglu I. Stern C. E. (2014). Structural differences in hippocampal and prefrontal gray matter volume support flexible context-dependent navigation ability. J. Neurosci. 34, 2314–2320. doi: 10.1523/JNEUROSCI.2202-13.2014, PMID: 24501370 PMC3913873

[ref10] Budhani S. Marsh A. A. Pine D. S. Blair R. J. R. (2007). Neural correlates of response reversal: considering acquisition. NeuroImage 34, 1754–1765. doi: 10.1016/j.neuroimage.2006.08.060, PMID: 17188518

[ref11] Cacciaglia R. Molinuevo J. L. Sánchez-Benavides G. Falcón C. Gramunt N. Brugulat-Serrat A.. (2018). Episodic memory and executive functions in cognitively healthy individuals display distinct neuroanatomical correlates which are differentially modulated by aging. Hum. Brain Mapp. 39, 4565–4579. doi: 10.1002/hbm.24306 29972619 PMC6220988

[ref12] Carlson J. M. Fang L. Koster E. H. W. Andrzejewski J. A. Gilbertson H. Elwell K. A.. (2022). Neuroplastic changes in anterior cingulate cortex gray matter volume and functional connectivity following attention bias modification in high trait anxious individuals. Biol. Psychol. 172:108353. doi: 10.1016/j.biopsycho.2022.108353 35569575

[ref13] Chaumon M. Kveraga K. Barrett L. F. Bar M. (2014). Visual predictions in the orbitofrontal cortex rely on associative content. Cereb. Cortex 24, 2899–2907. doi: 10.1093/cercor/bht146, PMID: 23771980 PMC4193460

[ref14] Clark I. A. Monk A. M. Hotchin V. Pizzamiglio G. Liefgreen A. Callaghan M. F.. (2020). Does hippocampal volume explain performance differences on hippocampal-dependant tasks? Neuroimage 221:117211. doi: 10.1016/j.neuroimage.2020.117211, PMID: 32739555 PMC7762813

[ref15] Collette F. Van Der Linden M. Delfiore G. Degueldre C. Luxen A. Salmon E. (2001). The functional anatomy of inhibition processes investigated with the Hayling task. Neuroimage 14, 258–267. doi: 10.1006/nimg.2001.0846, PMID: 11467901

[ref16] Duits P. Cath D. C. Heitland I. Baas J. M. P. (2016). High current anxiety symptoms, but not a past anxiety disorder diagnosis, are associated with impaired fear extinction. Front. Psychol. 7:252. doi: 10.3389/fpsyg.2016.00252, PMID: 26955364 PMC4767935

[ref17] Duits P. Cath D. C. Lissek S. Hox J. J. Hamm A. O. Engelhard I. M.. (2015). Updated meta-analysis of classical fear conditioning in the anxiety disorders. Depress. Anxiety 32, 239–253. doi: 10.1002/da.22353, PMID: 25703487

[ref18] Fullana M. A. Albajes-Eizagirre A. Soriano-Mas C. Vervliet B. Cardoner N. Benet O.. (2018). Fear extinction in the human brain: a meta-analysis of fMRI studies in healthy participants. Neurosci. Biobehav. Rev. 88, 16–25. doi: 10.1016/j.neubiorev.2018.03.002, PMID: 29530516

[ref19] Gonzalez S. T. Fanselow M. S. (2020). The role of the ventromedial prefrontal cortex and context in regulating fear learning and extinction. Psychol. Neurosci. 13, 459–472. doi: 10.1037/pne0000207 34504659 PMC8425341

[ref20] Hampshire A. Chamberlain S. R. Monti M. M. Duncan J. Owen A. M. (2010). The role of the right inferior frontal gyrus: inhibition and attentional control. NeuroImage 50, 1313–1319. doi: 10.1016/j.neuroimage.2009.12.109, PMID: 20056157 PMC2845804

[ref21] Hassabis D. Kumaran D. Maguire E. A. (2007). Using imagination to understand the neural basis of episodic memory. J. Neurosci. 27, 14365–14374. doi: 10.1523/JNEUROSCI.4549-07.2007, PMID: 18160644 PMC2571957

[ref22] Howard L. R. Kumaran D. Olafsdottir H. F. Spiers H. J. (2011). Double dissociation between hippocampal and Parahippocampal responses to object-background context and scene novelty. J. Neurosci. 31, 5253–5261. doi: 10.1523/JNEUROSCI.6055-10.2011, PMID: 21471360 PMC3079899

[ref23] Kalisch R. Korenfeld E. Stephan K. E. Weiskopf N. Seymour B. Dolan R. J. (2006). Context-dependent human extinction memory is mediated by a ventromedial prefrontal and hippocampal network. J. Neurosci. 26, 9503–9511. doi: 10.1523/JNEUROSCI.2021-06.2006, PMID: 16971534 PMC2634865

[ref24] Kannurpatti S. S. Motes M. A. Rypma B. Biswal B. B. (2010). Neural and vascular variability and the fMRI-BOLD response in normal aging. Magn. Reson. Imaging 28, 466–476. doi: 10.1016/j.mri.2009.12.007, PMID: 20117893 PMC2860003

[ref25] Klass A. Glaubitz B. Tegenthoff M. Lissek S. (2017). D-Cycloserine facilitates extinction learning and enhances extinction-related brain activation. Neurobiol. Learn. Mem. 144, 235–247. doi: 10.1016/j.nlm.2017.08.003, PMID: 28807795

[ref26] Klass A. Otto T. Tegenthoff M. Lissek S. (2021). The DA-antagonist tiapride affects context-related extinction learning in a predictive learning task, but not initial forming of associations, or renewal. Neurobiol. Learn. Mem. 183:107465. doi: 10.1016/j.nlm.2021.107465, PMID: 34015443

[ref27] Koch K. Reess T. J. Rus O. G. Zimmer C. (2016). Extensive learning is associated with gray matter changes in the right hippocampus. NeuroImage 125, 627–632. doi: 10.1016/j.neuroimage.2015.10.056, PMID: 26518629

[ref28] Koechlin E. Summerfield C. (2007). An information theoretical approach to prefrontal executive function. Trends Cogn. Sci. 11, 229–235. doi: 10.1016/j.tics.2007.04.005, PMID: 17475536

[ref29] Li R. Qin W. Zhang Y. Jiang T. Yu C. (2012). The neuronal correlates of digits backward are revealed by voxel-based morphometry and resting-state functional connectivity analyses. PLoS One 7:877. doi: 10.1371/journal.pone.0031877, PMID: 22359639 PMC3281094

[ref30] Lissek S. Glaubitz B. Klass A. Tegenthoff M. (2018). The effects of dopaminergic D2-like receptor stimulation upon behavioral and neural correlates of renewal depend on individual context processing propensities. Neuroimage 169, 69–79. doi: 10.1016/j.neuroimage.2017.12.022, PMID: 29242106

[ref31] Lissek S. Glaubitz B. Schmidt-Wilcke T. Tegenthoff M. (2016). Hippocampal context processing during acquisition of a predictive learning task is associated with renewal in extinction recall. J. Cogn. Neurosci. 28:928. doi: 10.1162/jocn_a_00928 26807840

[ref32] Lissek S. Glaubitz B. Uengoer M. Tegenthoff M. (2013). Hippocampal activation during extinction learning predicts occurrence of the renewal effect in extinction recall. Neuroimage 81, 131–143. doi: 10.1016/j.neuroimage.2013.05.025, PMID: 23684875

[ref33] Lissek S. Glaubitz B. Wolf O. T. Tegenthoff M. (2015). The DA antagonist tiapride impairs context-related extinction learning in a novel context without affecting renewal. Front. Behav. Neurosci. 9, 1–13. doi: 10.3389/fnbeh.2015.00238, PMID: 26388752 PMC4558976

[ref34] Lissek S. Klass A. Tegenthoff M. (2019). Effects of noradrenergic stimulation upon context-related extinction learning performance and BOLD activation in hippocampus and prefrontal cortex differ between participants showing and not showing renewal. Front. Behav. Neurosci. 13:78. doi: 10.3389/fnbeh.2019.00078, PMID: 31105536 PMC6491890

[ref35] Lissek S. Klass A. Tegenthoff M. (2020). Left inferior frontal gyrus participates in mediating the renewal effect irrespective of context salience. Front. Behav. Neurosci. 14, 1–16. doi: 10.3389/fnbeh.2020.00043, PMID: 32292332 PMC7118360

[ref36] Lissek S. Schlaffke L. Tegenthoff M. (2024). Microstructural properties of attention-related white matter tracts are associated with the renewal effect of extinction. Behav. Brain Res. 471:115125. doi: 10.1016/j.bbr.2024.115125, PMID: 38936425

[ref38] Lissek S. Tegenthoff M. (2024). Dissimilarities of neural representations of extinction trials are associated with extinction learning performance and renewal level. Front. Behav. Neurosci. 18:825. doi: 10.3389/fnbeh.2024.1307825, PMID: 38468709 PMC10925752

[ref39] Maguire E. A. Woollett K. Spiers H. J. (2006). London taxi drivers and bus drivers: a structural MRI and neuropsychological analysis. Hippocampus 16, 1091–1101. doi: 10.1002/hipo.20233, PMID: 17024677

[ref40] Matsuo K. Nicoletti M. Nemoto K. Hatch J. P. Peluso M. A. M. Nery F. G.. (2008). A voxel-based morphometry study of frontal gray matter correlates of impulsivity. Hum. Brain Mapp. 30:588. doi: 10.1002/hbm.20588, PMID: 18465751 PMC6870717

[ref41] Milad M. R. Quinn B. T. Pitman R. K. Orr S. P. Fischl B. Rauch S. L. (2005). Thickness of ventromedial prefrontal cortex in humans is correlated with extinction memory. Proc. Natl. Acad. Sci. USA 102, 10706–10711. doi: 10.1073/pnas.0502441102, PMID: 16024728 PMC1180773

[ref42] Milad M. R. Wright C. I. Orr S. P. Pitman R. K. Quirk G. J. Rauch S. L. (2007). Recall of fear extinction in humans activates the ventromedial prefrontal cortex and hippocampus in concert. Biol. Psychiatry 62, 446–454. doi: 10.1016/j.biopsych.2006.10.011, PMID: 17217927

[ref43] Mitchell D. G. V. Luo Q. Avny S. B. Kasprzycki T. Gupta K. Chen G.. (2009). Adapting to dynamic stimulus-response values: differential contributions of inferior frontal, dorsomedial, and dorsolateral regions of prefrontal cortex to decision making. J. Neurosci. 29, 10827–10834. doi: 10.1523/JNEUROSCI.0963-09.2009, PMID: 19726640 PMC2774778

[ref44] Mystkowski J. L. Craske M. G. Echiverri A. M. (2002). Treatment context and return of fear in spider phobia. Behav. Ther. 33, 399–416. doi: 10.1016/S0005-7894(02)80035-1 16942960

[ref45] Poppenk J. McIntosh A. R. Craik F. I. M. Moscovitch M. (2010). Past experience modulates the neural mechanisms of episodic memory formation. J. Neurosci. 30, 4707–4716. doi: 10.1523/JNEUROSCI.5466-09.2010, PMID: 20357121 PMC6632301

[ref46] Ranganath C. Johnson M. K. D’Esposito M. (2000). Left anterior prefrontal activation increases with demands to recall specific perceptual information. J. Neurosci. 20, 1–5. doi: 10.1523/JNEUROSCI.20-22-j0005.2000 11069977 PMC6773176

[ref47] Rubia K. Smith A. B. Brammer M. J. Taylor E. (2003). Right inferior prefrontal cortex mediates response inhibition while mesial prefrontal cortex is responsible for error detection. Neuroimage 20, 351–358. doi: 10.1016/S1053-8119(03)00275-1, PMID: 14527595

[ref48] Sehlmeyer C. Schöning S. Zwitserlood P. Pfleiderer B. Kircher T. Arolt V.. (2009). Human fear conditioning and extinction in neuroimaging: a systematic review. PLoS One 4:e5865. doi: 10.1371/journal.pone.0005865, PMID: 19517024 PMC2692002

[ref49] Smolker H. R. Depue B. E. Reineberg A. E. Orr J. M. Banich M. T. (2015). Individual differences in regional prefrontal gray matter morphometry and fractional anisotropy are associated with different constructs of executive function. Brain Struct. Funct. 220, 1291–1306. doi: 10.1007/s00429-014-0723-y, PMID: 24562372 PMC4320016

[ref50] Sun J. Chen Q. Zhang Q. Li Y. Li H. Wei D.. (2016). Training your brain to be more creative: brain functional and structural changes induced by divergent thinking training. Hum. Brain Mapp. 37, 3375–3387. doi: 10.1002/hbm.23246, PMID: 27159407 PMC6867508

[ref51] Swick D. Ashley V. Turken A. U. (2008). Left inferior frontal gyrus is critical for response inhibition. BMC Neurosci. 9, 1–11. doi: 10.1186/1471-2202-9-102, PMID: 18939997 PMC2588614

[ref52] Takeuchi H. Taki Y. Sassa Y. Sekiguchi A. Nagase T. Nouchi R.. (2015). The associations between regional gray matter structural changes and changes of cognitive performance in control groups of intervention studies. Front. Hum. Neurosci. 9:681. doi: 10.3389/fnhum.2015.00681, PMID: 26733852 PMC4685061

[ref53] Ting C. C. Wu S. W. Wu S. W. Yu C. C. Maloney L. T. Maloney L. T.. (2015). Neural mechanisms for integrating prior knowledge and likelihood in value-based probabilistic inference. J. Neurosci. 35, 1792–1805. doi: 10.1523/JNEUROSCI.3161-14.2015, PMID: 25632152 PMC4308614

[ref54] Tzourio-Mazoyer N. Landeau B. Papathanassiou D. Crivello F. Etard O. Delcroix N.. (2002). Automated anatomical labeling of activations in SPM using a macroscopic anatomical parcellation of the MNI MRI single-subject brain. NeuroImage 15, 273–289. doi: 10.1006/nimg.2001.0978, PMID: 11771995

[ref55] Uengoer M. Klass A. Tegenthoff M. Lissek S. (2020). Test-retest reliability of response recovery after discrimination reversal learning. Behav. Process. 176:104107. doi: 10.1016/j.beproc.2020.104107, PMID: 32348808

[ref56] Üngör M. Lachnit H. (2006). Contextual control in discrimination reversal learning. J. Exp. Psychol. Anim. Behav. Process. 32, 441–453. doi: 10.1037/0097-7403.32.4.441, PMID: 17044746

[ref57] Van Petten C. (2004). Relationship between hippocampal volume and memory ability in healthy individuals across the lifespan: review and meta-analysis. Neuropsychologia 42, 1394–1413. doi: 10.1016/j.neuropsychologia.2004.04.006, PMID: 15193947

[ref58] Vassena E. Holroyd C. B. Alexander W. H. (2017). Computational models of anterior cingulate cortex: at the crossroads between prediction and effort. Front. Neurosci. 11:316. doi: 10.3389/fnins.2017.00316, PMID: 28634438 PMC5459890

[ref59] Wang Q. Chen C. Cai Y. Li S. Zhao X. Zheng L.. (2016). Dissociated neural substrates underlying impulsive choice and impulsive action. NeuroImage 134, 540–549. doi: 10.1016/j.neuroimage.2016.04.010, PMID: 27083527

[ref60] Winkelmann T. Grimm O. Pohlack S. T. Nees F. Cacciaglia R. Dinu-Biringer R.. (2016). Brain morphology correlates of interindividual differences in conditioned fear acquisition and extinction learning. Brain Struct. Funct. 221, 1927–1937. doi: 10.1007/s00429-015-1013-z, PMID: 25716297

[ref61] Woollett K. Glensman J. Maguire E. A. (2008). Non-spatial expertise and hippocampal gray matter volume in humans. Hippocampus 18, 981–984. doi: 10.1002/hipo.20465, PMID: 18566963 PMC2572201

[ref62] Yuan P. Raz N. (2014). Prefrontal cortex and executive functions in healthy adults: a meta-analysis of structural neuroimaging studies. Neurosci. Biobehav. Rev. 42, 180–192. doi: 10.1016/j.neubiorev.2014.02.005, PMID: 24568942 PMC4011981

[ref63] Zeidman P. Lutti A. Maguire E. A. (2015). Investigating the functions of subregions within anterior hippocampus. Cortex 73, 240–256. doi: 10.1016/j.cortex.2015.09.002, PMID: 26478961 PMC4686003

[ref64] Zhang Z. Mendelsohn A. Manson K. F. Schiller D. Levy I. (2015). Dissociating value representation and inhibition of inappropriate affective response during reversal learning in the ventromedial prefrontal cortex. eNeuro 2, 1–16. doi: 10.1523/ENEURO.0072-15.2015 PMC4698540 26730406

